# A rare case of rapidly enlarging tracheal lobular capillary hemangioma presenting as difficult to ventilate acute asthma during pregnancy

**DOI:** 10.1186/1471-2466-14-41

**Published:** 2014-03-10

**Authors:** Shivesh Prakash, Shailesh Bihari, Ubbo Wiersema

**Affiliations:** 1 Department of Intensive care, Flinders Medical Centre, Bedford Park, 5042 South Australia, Australia; 2 Department of Critical Care Medicine, Flinders University, Bedford Park, 5042 South Australia, Australia

**Keywords:** Lobular Capillary Hemangioma, Asthma, Pregnancy, Veno-venous extra-corporeal membrane oxygenation

## Abstract

**Background:**

Lobular Capillary Hemangioma (LCH) is a benign tumour that is known to be hormone responsive and have a relatively high incidence during pregnancy, the most common site being the gingival surfaces. A tracheal origin for this tumour is extremely rare, with no case reported so far in this patient population, and the only reported clinical presentation of tracheal LCH in the literature is with haemoptysis.

**Case presentation:**

We describe a case of a 23-year-old known asthmatic who presented at 32 weeks gestation with life-threatening respiratory failure resembling acute severe asthma, requiring invasive ventilation which was extremely difficult. This was subsequently found to be due to a large tracheal LCH producing a ball-valve phenomenon and predominantly expiratory airflow limitation similar to acute asthma. The endotracheal tube was advanced past the lesion under bronchoscopic guidance, and urgent Caesarean section performed due to foetal distress. The tumour was subsequently debulked and the trachea stented, facilitated by bi-femoral veno-venous extra-corporeal membrane oxygenation with relatively low dose of heparin.

**Conclusion:**

To our knowledge, this is the first report of a unique presentation and management of largest tracheal LCH so far occurring during pregnancy. Pulmonary and critical care physicians should be aware of this unique differential of refractory asthma, the aggressive nature of this benign tumour due to hormonal influences during pregnancy, and feasibility of using bi-femoral veno-venous extra-corporeal membrane oxygenation with low dose heparin as a rescue, given the high risk of bleeding.

## Background

Primary tracheal tumours are rare with an estimated incidence of about 2.7 new cases per million per year and are usually malignant in adults [1]. Lobular Capillary Haemangioma (LCH; pyogenic granuloma) is a benign tumour with a distinctive lobular arrangement of capillaries [2]. The usual sites for this tumour are the skin and the nasopharyngeal and oral mucosal surfaces. A tracheal origin for this tumour is, however, exceedingly rare, with the literature limited to only a few case reports [3,4]. Hence, little is known about the presentation, behaviour, and management of tracheal LCH. This tumour is hormone responsive and consequently has a relatively higher incidence, recurrence, and growth rate during pregnancy [5]. However, a tracheal origin for LCH has not been reported in this patient population so far. Also, none of the case reports have clearly revealed the recurrent nature and rate of growth of tracheal LCH. We present the case of a 23-year-old pregnant woman with asthma who presented with tracheal LCH masquerading as acute asthma, posing both a diagnostic and therapeutic challenge.

## Case presentation

A 32 weeks pregnant, 23-year-old known asthmatic woman presented to a peripheral hospital with acute respiratory failure during winter. Cold air was a known precipitant for her asthma. She was intubated and ventilated for presumed severe acute asthma with refractory bronchospasm. Following intubation she was found to be extremely difficult to ventilate and was retrieved to our intensive care unit for further management.

**Figure 1:**
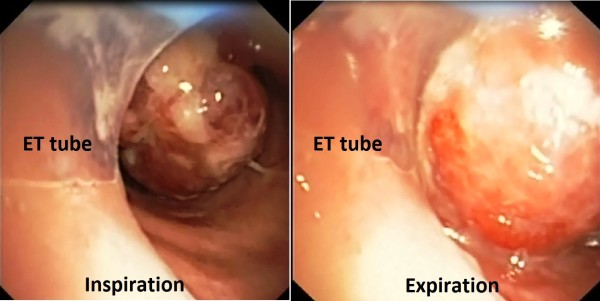
**Bronchoscopic view at the edge of endotracheal tube.** A highly vascular polypoidal lesion is seen, which causes a ball-valve phenomenon during respiration to generate severe compromise of expiratory airflow (Additional file 1: Video 1).

She had widespread, faint, prolonged monophonic wheeze on auscultation with no evidence of pneumothorax. Mechanical ventilation in volume control mode was commenced with an inspired oxygen fraction of 1.0, external Positive End Expiratory Pressure (PEEP) 0 cmH_2_O, respiratory rate six breaths per minute, a tidal volume of 300 ml, and inspiratory flow rate of 30 L/min. However, high peak airway pressures truncated each breath. Intrinsic PEEP was measured as 30 cmH_2_O, with evidence of dynamic hyperinflation and accompanying hemodynamic instability despite inspiratory to expiratory ratio of close to 1:16. An arterial blood gas sample revealed severe respiratory acidosis (pH of 6.92, PaO_2_ 116 mmHg, and PaCO_2_ 143 mmHg). There was evidence of foetal distress on Cardiotocograph (CTG). Bronchoscopy demonstrated a pedunculated tumour arising from the posterior tracheal wall, extending from the tip of the Endotracheal Tube (ET) and measuring approximately 4 cm by 2 cm. The tumour acted as a ball valve to cause severe expiratory airflow obstruction (Figure 1; Additional file 1: Video 1). The bronchoscope was advanced beyond the lesion and the ET advanced over the bronchoscope to bridge the lesion, resulting in dramatic improvements in lung mechanics and haemodynamics. Urgent Caesarean section was performed due to persistent decelerations on CTG, with birth of a healthy baby. Access to her previous records revealed that she previously presented with haemoptysis at 16 weeks of the current gestation. At this time she had a small polyp (~0.5 cm) in the same tracheal location, which had been excised and diagnosed as LCH.

**Figure 2:**
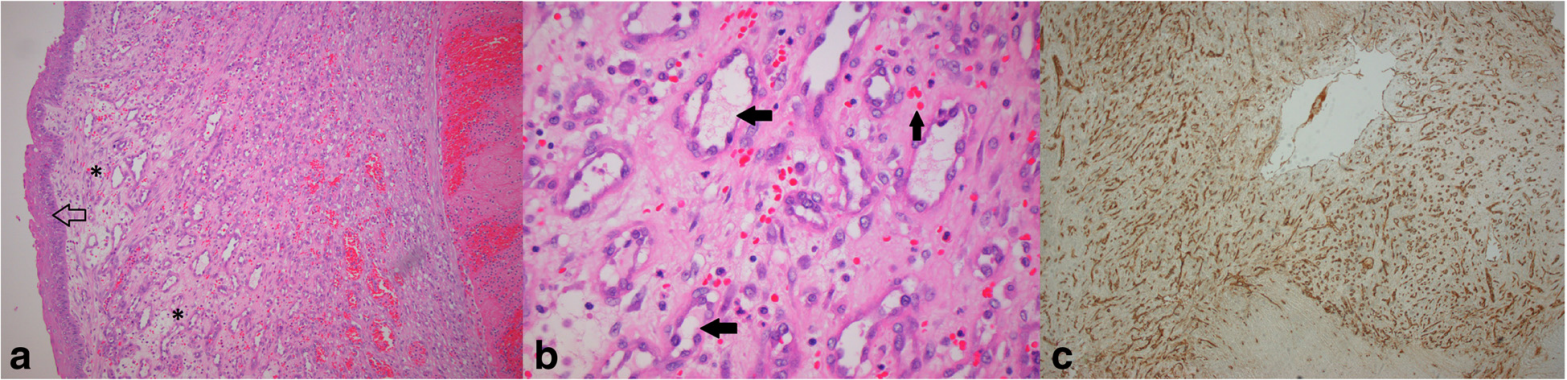
**Histopathological features of Lobular capillary hemangioma.** Hematoxylin and eosin stained sections at **(a)** x100 Magnification and **(b)** x400 Magnification, and **(c)** CD31 Immunohistochemical staining, (Magnification x40).

The following day, tumour debulking was attempted. In view of the high risk of major haemorrhage and hypoxemia, precautionary cannulation for veno-venous Extra-Corporeal Membrane Oxygenation (ECMO) was established via a bi-femoral approach. Surgical debulking was complicated by significant haemorrhage causing desaturation (SpO_2_ < 80%). She was promptly commenced on ECMO with recovery of arterial oxygen saturation and minimal disruption to surgery. A small bolus of heparin (2000 units) was given intravenously just prior to connection of the ECMO circuit, but no further heparin was administered until after surgery. On return to the ICU, a low dose heparin infusion (10 units/kg/hour) was used to maintain ECMO circuit patency. The histopathology revealed recurrent LCH (Figure 2), with intact overlying epithelium (Arrow) and lobular arrangements (*) of proliferating capillaries (horizontal arrow) in an edematous fibro-myxoid stroma, containing extravasated red blood cells (vertical arrow). There was intense staining with CD31 Immunohistochemical staining highlighting proliferating endothelial cells and vascular origin of tumor. Her trachea was subsequently stented and ECMO was discontinued. She made a full recovery.

## Discussion

Obstructing airway lesions are recognised in the differential diagnosis of refractory asthma. Primary tracheal tumours are rare with an estimated incidence of about 2.7 new cases per million per year and are usually malignant in adults [1]. Amongst all tracheal tumours, LCH is exceedingly rare with only few case reports in the literature [3,4]. The usual sites for this tumour are the skin and the nasopharyngeal and oral mucosae. The cause of tracheal obstruction in this case was found to be a large LCH. To our knowledge, this is the largest case of tracheal LCH reported to date. LCH occurs in patients of all ages with a peak incidence in the second and third decades of life [6]. Mucosal LCH is more common in adult women than in men or children [7].

Microscopically, LCH has a distinctive lobular arrangement of capillaries of varying calibre embedded in a fibromyxoid matrix without atypical mitoses [2]. Conventional granulation tissue is the most important histological differential diagnosis. Since LCH is most commonly found in locations prone to trauma such as skin, gingiva, nail beds, etc., finding ulceration and coexisting chronic inflammation in the stroma is fairly common. When inflammation is marked, the overall features show a close resemblance to granulation tissue, except for the presence of capillary lobules in the deeper dermis at the base of the lesion, lobular arrangements of capillaries instead of radial, and proliferating endothelial cells on immunohistochemical staining (e.g., with anti-CD31 antibodies in this case). The granulomatous appearance is highly variable depending on the location of the tumour and is frequent in cutaneous and mucosal locations, which are more prone to trauma. Besides histology, LCH differs from granulation tissue by its rapid growth, multiple occurrence, and frequent recurrence. The lesion tends to have a pedunculated macroscopic morphology, which along with its large size led to the ball-valve phenomenon and phasic airflow limitation in this case [2]. The pedunculated morphology is seen in most LCH occurring on the skin but only in 30 percent of mucosal LCH, mainly in those involving the oral mucosa [2]. Such a presentation of LCH has not been reported before. The first presentation, however, was with haemoptysis and this is the most typical presentation apart from simple cough that has been reported in the literature.

The pathogenesis of this lesion is currently uncertain. Most theories on pathogenesis revolve around LCH as a hyperplastic, neovascular response to an angiogenic stimulus with imbalance of promoters and inhibitors [8]. Angiogenic growth factors such as vascular endothelial growth factor (VEGF) and decorin, transcription factors (pATF2 and pSTAT3), and signal transduction pathways (MAPK) are overexpressed in LCH, but their exact role is undetermined [9,10]. There is a reported association between LCH and pregnancy (5% incidence), where it is termed granuloma gravidarum [5]. LCH are known to recur and grow rapidly during pregnancy, presumably due to responsiveness to progesterone, which appears to act via stimulation of angiogenic factors and inhibition of apoptosis [5,11]. This may explain the recurrent nature of the tumour and the rapidity of growth in the case discussed. Trauma has been reported as a trigger, although only 7 to 23 % of patients with LCH report a previous injury at the site [12]. There was no history of airway instrumentation or foreign body aspiration prior to the bronchoscopy performed earlier in her gestation, when the tumour was already *in situ*.

The gingival surface is the most common site of LCH during pregnancy, with no cases of tracheal LCH reported during pregnancy to date. None of the case reports have clearly revealed the recurrent nature and rate of growth of tracheal LCH as they were already large, symptomatic, and requiring treatment at the time of diagnosis. The current knowledge about these features of this tumour mainly originates from literature on extra-tracheal sites of LCH, particularly the skin. LCH rarely exceeds 2.5 cm in size and it usually reaches its full size within weeks or months, remaining static thereafter [13]. The LCH occurring in pregnant women usually regress after childbirth over a period of 6 to 18 months. A recurrence rate of 16% has been reported for oral LCH and is believed to result from incomplete excision, failure to remove etiological factors, or re-injury of the area [14]; the inability to remove aetiological factors is probably responsible for the higher recurrence rate observed during pregnancy. The case discussed is the first evidence to suggest that tracheal LCH can have high recurrence rate and rapid growth, justifying surveillance after initial treatment.

The mainstay of treatment, if symptomatic, is surgical debulking or excision using various methods [2-4,12]. The use of ECMO to facilitate tracheal surgery has previously been reported [15], and this case illustrates the feasibility of low dose heparinisation and bifemoral cannulation, which facilitated the rapid use of ECMO with minimal interruption to surgery. ECMO has not been utilised in the management in the previous case reports of tracheal LCH. ECMO has significant risks and cost implications and is not recommended as routine in every case of tracheal LCH. A few characteristics of this particular case, however, prompted consideration of use of ECMO, such as the large size of the tumour, its highly vascular nature due to the hormonal influence of pregnancy, and its proximity to the carina. Even then, ECMO was planned as a rescue and not as a routine first-line strategy to facilitate surgery.

## Conclusion

Here we describe a rare differential of refractory asthma - the largest LCH tumour reported to date, occurring at an exceedingly rare site and with rapid growth during pregnancy, possibly due to hormonal influence. Pulmonary and critical care physicians should be aware of this unique differential of refractory asthma, the association with pregnancy, and the feasibility of using bi-femoral veno-venous ECMO with low-dose heparin for its management.

### Consent

Written informed consent was obtained from the patient for publication of this case report and any accompanying images. A copy of the written consent is available for review by the Editor of this journal.

## Abbreviations

CTG: Cardiotocograph; ET: Endotracheal tube; ECMO: Extra-corporeal membrane oxygenation; LCH: Lobular capillary hemangioma; PEEP: Positive end expiratory pressure.

## Competing interests

The authors declare that they have no competing interests.

## Authors’ contributions

SP and SB analysed and interpreted the patient’s data and drafted the manuscript. UW revised the clinical data and supervised the case report. All authors read and approved the final manuscript.

## Authors’ information

SP and SB have MD in pulmonary medicine and currently hold Australian fellowship in intensive care. Their area of interest includes critical care aspects of pulmonary medicine. UW is a senior consultant in intensive care. He has expertise in the area of ECMO and has been the director of ECMO services at Flinders Medical Centre Intensive care.

## Pre-publication history

The pre-publication history for this paper can be accessed here:

http://www.biomedcentral.com/1471-2466/14/41/prepub

## Supplementary Material

Additional file 1 **Video 1.** Bronchoscopic view at the edge of endotracheal tube. A highly vascular polypoidal lesion is seen, which causes a ball-valve phenomenon during respiration to generate severe compromise of expiratory airflow. Click here for file

## References

[B1] Lau CL Patterson GA Flint PW Diagnosis and Management of Tracheal Neoplasms Cummings Otolaryngology Head and Neck Surgery 2005 4 PA: Elsevier Mosby 2478 2480

[B2] Mills SE Cooper PH Fechner RE Lobular capillary hemangioma: the underlying lesion of pyogenic granuloma; a study of 73 cases from the oral and nasal mucous membranes Am J Surg Pathol 1980 4 470 479 7435775

[B3] Irani S Brack T Pfaltz M Russi EW Tracheal lobular capillary hemangioma: a rare case of recurrent hemoptysis Chest 2003 123 2148e9 12796203 10.1378/chest.123.6.2148

[B4] Amy FT Enrique DG Lobular Capillary Hemangioma in the Posterior Trachea: A Rare Cause of Hemoptysis [http://dx.doi.org/10.1155/2012/592524] 10.1155/2012/592524 PMC3514818 23227409

[B5] Sills ES Zegarelli DJ Hoschander MM Strider WE Clinical diagnosis and management of hormonally responsive oral pregnancy tumor (pyogenic granuloma) J Reprod Med 1996 41 467 470 8829057

[B6] Harris MN Desai R Chuang TY Hood AF Mirowski GW Lobular capillary hemangiomas: an epidemiologic report, with emphasis on cutaneous lesions J Am Acad Dermatol 2000 42 1012 10.1067/mjd.2000.104520 10827405

[B7] Gordón-Núñez MA De Vasconcelos Carvalho M Benevenuto TG Lopes MF Silva LM Galvão HC Oral pyogenic granuloma: a retrospective analysis of 293 cases in a Brazilian population J Oral Maxillofac Surg 2010 68 2185 10.1016/j.joms.2009.07.070 20417014

[B8] Giblin AV Clover AJ Athanassopoulos A Budny PG Pyogenic granuloma - the quest for optimum treatment: audit of treatment of 408 cases J Plast Reconstr Aesthet Surg 2007 60 1030 10.1016/j.bjps.2006.10.018 17478135

[B9] Chen SY Takeuchi S Urabe K Hayashida S Kido M Tomoeda H Uchi H Dainichi T Takahara M Shibata S Tu YT Furue M Moroi Y Overexpression of phosphorylated-ATF2 and STAT3 in cutaneous angiosarcoma and pyogenic granuloma J Cutan Pathol 2008 35 722 10.1111/j.1600-0560.2007.00887.x 18700251

[B10] Arbiser JL Weiss SW Arbiser ZK Bravo F Govindajaran B Caceres-Rios H Cotsonis G Recavarren S Swerlick RA Cohen C Differential expression of active mitogen-activated protein kinase in cutaneous endothelial neoplasms: implications for biologic behavior and response to therapy J Am Acad Dermatol 2001 44 193 10.1067/mjd.2000.111632 11174372

[B11] Yuan K Wing LY Lin MT Pathogenetic roles of angiogenic factors in pyogenic granulomas in pregnancy are modulated by female sex hormones J Periodontol 2002 73 701 708 10.1902/jop.2002.73.7.701 12146528

[B12] Patrice SJ Wiss K Mulliken JB Pyogenic granuloma (lobular capillary hemangioma): a clinicopathologic study of 178 cases Pediatr Dermatol 1991 8 267 10.1111/j.1525-1470.1991.tb00931.x 1792196

[B13] Bouquot JE Nikai H Gnepp DR Lesions of the Oral Cavity Diagnostic Surgical Pathology of the Head and Neck 2001 1 Philadelphia: WB Saunders 141 233

[B14] Taira JW Hill TL Everett MA Lobular capillary hemangioma (pyogenic granuloma) with satellitosis J Am Acad Dermatol 1992 27 297 300 10.1016/0190-9622(92)70184-H 1517491

[B15] Smith IJ Sidebotham DA McGeorge AD Dorman EB Wilsher ML Kolbe J Use of extracorporeal membrane oxygenation during resection of tracheal papillomatosis Anesthesiology 2009 110 427 429 19194169 10.1097/ALN.0b013e3181943288

